# Impact of a *Lactobacillus* dominant cervical microbiome, based on 16S-FAST profiling, on the reproductive outcomes of IVF patients

**DOI:** 10.3389/fcimb.2023.1059339

**Published:** 2023-05-26

**Authors:** Wenzheng Guan, Sitong Dong, Zhen Wang, Jiao Jiao, Xiuxia Wang

**Affiliations:** ^1^ Center of Reproductive Medicine, Shengjing Hospital of China Medical University, Shenyang, China; ^2^ Shenyang Reproductive Health Clinical Medicine Research Center, Shenyang, China; ^3^ Key Laboratory of Reproductive and Genetic Medicine, China Medical University, National Health Commission, Shenyang, China

**Keywords:** cervical microbiome, *Lactobacillus crispatus*, reproductive outcomes, frozen embryo transfer, 16S full-length assembly sequencing technology

## Abstract

**Objective:**

This study assessed the impact of the cervical microbiome on reproductive outcomes in frozen embryo transfer (FET) patients.

**Study design:**

This cross-sectional study included 120 women (aged 20–40 years) undergoing FET. A cervical sample obtained before embryo transfer was analyzed using 16S full-length assembly sequencing technology (16S-FAST), which detects full length 16S rDNA.

**Results:**

We found that >48% of the identified *Lactobacillus* species were novel. The cervical microbiome was clustered into three cervical microbiome types (CMT): CMT1, dominated by *L. crispatus*; CMT2, dominated by *L. iners*; and CMT3, dominated by other bacteria. CMT1 had a significantly higher biochemical pregnancy rate (*P*=0.008) and clinical pregnancy rate (*P*=0.006) than CMT2 and CMT3. Logistic analysis showed that compared to CMT1, CMT2 and CMT3 were independent risk factors for biochemical pregnancy failure (odds ratio [OR]: 6.315, 95% confidence interval [CI]: 2.047-19.476, *P*=0.001; OR: 3.635, 95% CI: 1.084-12.189, *P*=0.037) and clinical pregnancy failure (OR: 4.883, 95% CI: 1.847-12.908, *P*=0.001; OR: 3.478, 95% CI: 1.221-9.911, *P*=0.020). A *L. crispatus*-dominated group as a diagnostic indicator of biochemical and clinical pregnancy positive had area under the curve (AUC) values of 0.651(*P*=0.008) and 0.645(*P*=0.007), respectively. Combining the cervical microbiome with embryonic stage optimized the diagnostic performance for biochemical and clinical pregnancy failure with AUC values of 0.743(*P*<0.001) and 0.702(*P*<0.001), respectively. Additionally, relative abundance of *L. crispatus* predicted biochemical pregnancy positive with AUC values of 0.679(*P*=0.002) and clinical pregnancy positive with AUC values of 0.659(*P*=0.003).

**Conclusion:**

Cervical microbiome profiling using 16S-FAST enables stratification of the chance of becoming pregnant prior to FET. Knowledge of the cervical microbiota may enable couples to make more balanced decisions regarding the timing and continuation of FET treatment cycles.

## Introduction

Infertility affects 8–12% of reproductive-aged couples worldwide (Inhorn and Patrizio, 2015). Common causes of infertility include ovulatory disorders, tubal disease, inflammation, and sperm abnormalities; however, one-third of people are infertile due to unknown reasons (Bashiri et al., 2018; Carson and Kallen, 2021). *In vitro* fertilization and embryo transfer (IVF-ET) are currently used as effective treatments for infertility. However, the pregnancy rate remains approximately 50% (Zhang et al., 2021). Therefore, predicting the outcomes prior to the start of treatments may help to develop personalized treatment plans and reduce the psychological and financial stress for patients.

Female reproductive tract microbiota such as vaginal, cervical and uterus microbiota, may affect pregnancy outcome in IVF patients (Chen et al., 2017). A study showed that vaginal microbial community could not differ clinical pregnancy in *in vitro* fertilization-embryo transfer (IVF-ET) (Wang et al., 2021). While Haahr’ study showed that qPCR defined bacterial vaginosis (BV) associated abnormal vaginal microbiota was correlated to clinical pregnancy negative(Haahr et al., 2016). Microbiota of cervix which is in the transition zone between the vagina and uterus, is now getting increasing attention. A few studies have investigated the cervical bacteria and IVF pregnancy outcomes (Hao et al., 2021; Wang et al., 2021; Villani et al., 2022), but the effects of prediction were inconsistent. Although it seems the reproductive tract microbiota may have a negative impact on embryo implantation in biologically plausible, more research is needed in this field.

*Lactobacillus* is the most common and significant factor in the stabilization and imbalance of reproductive tract microbiome (Gajer et al., 2012; Ravel et al., 2013). The evidence of relationship between *Lactobacillus* and pregnancy outcome is conflicting. *Lactobacillus* was reported to significantly decreased in the infertile patients (Fu et al., 2020; Zhao et al., 2020; Karaer et al., 2021; Sezer et al., 2022), and women achieved biochemical pregnancy had higher level of *Lactobacillus* spp. (Bernabeu et al., 2019). However, another study reported that *Lactobacillus* showed no difference between women who got clinical pregnancy or not (Wang et al., 2021). It is supposed that the different results might be induced by insufficient identification of bacterial species levels. For instance, women with *L. iners* dominated microbiota were more susceptible to vaginal dysbiosis, bacterial vaginosis (BV) and sexually transmitted infections than *L. crisptus* (Borgdorff et al., 2014; Jespers et al., 2015; Reimers et al., 2016; van Houdt et al., 2018; Witkin et al., 2021). Thus, the effect of reproductive microbiota on pregnancy outcome might require further distinction of species level.

Until now, a few studies have used full-length of 16S rRNA sequence analysis to describe feature of microbiome. Full-length of 16S rRNA sequence analysis was reported to catalog the diversity of deeply branching phyla and identify species (Karst et al., 2018). Our previous study used 16S rDNA full-length assembly sequencing technology (16S-FAST) (Dong et al., 2021) also found improved discrimination ability on species level, because it reads the entire variable region of the 16S rRNA gene (V1-V9). In the current article, we aim to use 16S-FAST to describe the characteristics of cervical microbiota, and evaluate performance of 16S-FAST in predicting the biochemical and clinical pregnancy outcome of IVF treatment.

## Materials and methods

### Study population

From September 2021 to December 2021, 230 planned FETs were performed. The inclusion criteria were as follows: female aged 20 to 40 years; good-quality cleavage-stage embryo or blastocysts transferred; endometrial thickness >8 mm; hormone replacement therapy (HRT) protocols; ethnic group is Han. The exclusion criteria were as follows: more than three previous unsuccessful FETs; uterine malformation, intrauterine adhesions, uterine fibroids, adenomyosis, or endometritis; had taken probiotics or antibiotics within 1 month; smokers or alcoholics; participation in any experimental drug study within 60 days. Finally, 120 participants were enrolled (**Supplementary Figure 1**). In accordance with the Declaration of Helsinki, the design of the present study was approved by the Ethics Committee of the Shengjing Hospital of China Medical University (Reference No. 2021PS016F). All participants gave written informed consents.

### Endometrial preparation protocols and cervical sample collection

The endometrium was prepared with estradiol, which was administered *via* oral (titrated up to 2–3 mg twice per day) or transvaginal (titrated up to 1–2 mg twice per day) routes. Transvaginal ultrasound was performed to assess the endometrium after 12–14 days of estradiol administration, and progesterone was initiated once the endometrial lining was ≥8 mm and trilaminar in appearance. Progesterone (80 mg daily) was used for endometrial transformation. Cervical samples were collected before endometrium transformation according to the following steps: 1) a sterile cotton swab was placed into the patient’s cervical canal and rotated to obtain cervical samples, during this process, the operator ensured that the cotton swab did not touch the vaginal wall of the patient; 2) the samples were directly placed into the DNA storage tubes (CW2654, CwBiotech, Beijing, China).

### 16S full-length library construction technology

The cervical samples were stored at room temperature and sent to a laboratory within two hours. Storage buffer contains 100 mM Tris–HCl (pH 9), 40 mM EDTA, 4 M guanidine thiocyanate (protein denaturant to inhibit bacterial growth), and 0.001% bromothymol, as previously described (Hisada et al., 2015; Nishimoto et al., 2016). Bacterial DNA was extracted using a DNA extraction kit (Qiagen Fecal DNA Extraction Kit, Qiagen, Hilden, Germany). Quantitative and qualitative analyses as well as quality control of the extracted DNA were performed. Sequencing was performed *via* the methods described in our previous study (Dong et al., 2021).

### Bioinformatics analysis and statistical methods

16S rDNA full-length sequence was generated *via* the methods previously described (Dong et al., 2021). Operational taxonomic unit (OTU) was clustered with a similarity threshold of 99%. Species annotations for all OTUs were performed with SILVA_132_SSURef_Nr99 database with default parameters (Quast et al., 2013). The software used is Mothur (Schloss et al., 2009), and the name of the method is wang. Muscle 3.8.31 (Edgar, 2004) was used to perform multiple sequence alignment, and FastTree 2.1.11 (Price et al., 2009; Price et al., 2010) was used to construct the evolutionary tree. Clustering of communities based on community composition and abundance was performed using complete linkage hierarchical clustering with three clusters in the R package, and R package heatmap 1.0.12 was used to draw the heatmap. The α bacterial diversity of the cervical microbiota community was estimated by QIIME1 V1.8.0 (Caporaso et al., 2010). The difference between groups was calculated by a Kruskal–Wallis sum-rank tests. Principal coordinates analysis (PCoA) was performed using the R 3.6.1 package vegan 2.5–3 analysis, and the *P* value was calculated by matching the Adonis method. Kruskal–Wallis sum-rank tests were used to detect significant differences between groups. To assess the impact of significant variables on pregnancy outcomes, univariate and multivariate logistic regression analyses were conducted using SPSS statistics (version 23.0; IBM, Armonk, NY, USA) to determine the odds ratio (OR) and confidence interval (CI) for the presence of pregnancy negative, then age and embryonic stage were also included into analysis as covariates considering that they are also important factors affecting pregnancy outcomes. The receiver operating characteristic (ROC) curve was modeled using GraphPad Prism 6 (GraphPad Software).

### Reproductive outcomes measures

The reproductive outcomes were biochemical pregnancy rate (BPR) and clinical pregnancy rate (CPR). Biochemical pregnancy was defined as a human chorionic gonadotrophin (hCG) level ≥5 mIU/mL, and clinical pregnancy was defined as a gestational sac revealed by ultrasonography on the 21st day after a successful biochemical pregnancy.

## Results

### 16S rDNA FAST analysis of the cervical microbiome

**Figure 1:**
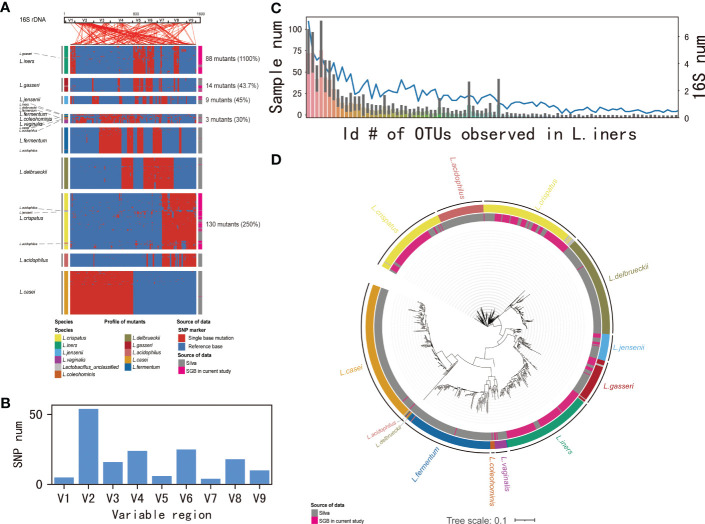
Identification of species in *Lactobacillus* by 16S-FAST. **(A)** Intra-species variation in *Lactobacillus* 16S rDNA genes. From left to right, 16S rDNA sequences annotated in the database, mutations detected in consensus sequences, and sources of reference data. Intra-species variations are clustered according to a presence/absence matrix. Coordinate positions in 16S rDNA are connected by lines above the panel. **(B)** SNP numbers in the variable region 1–9. **(C)** Numbers of samples that novel *L.iners* OTUs being observed. **(D)** Evolutionary tree showed detected 16S rDNA sequences annotated in the database and sources of reference data. Branch relations are connected by lines.

One-hundred-and-twenty samples containing more than 2000 contigs were filtered after sequencing full-length 16S rDNA. The rarefaction curve appeared flat, indicating that the amount of sequencing data was reasonable (**Supplementary Figure 2**). Consistent with previous reports, *Lactobacillus* was the most abundant bacterium in these samples (**Supplementary Figure 3**). *Lactobacillus* identified in cervical samples was from nine lineages, which was present as 796 unique *Lactobacillus* 16S rDNA operational taxonomic units (OTU)s with at least a 1% genetic difference. We noticed that the majority of OTUs were derived from five lineages: *L. iners, L. gasseri, L. jensenii, L. vaginalis*, and *L. crispatus*, displaying 1100%, 43.7%, 45%, 30%, and 250% of the novel sequences, respectively; Lineage *L. vaginalis* was also composed of several strains previously annotated as *L. iners, L. delbrueckii, L. fermentum, L. coleohominis, L. casei*, and *L. acidophilus*. Similar phenomena were observed in the other two lineages, *L. iners* (comprising *L. gasseri*) and *L*. *crispatus* (comprising *L. acidophilus* and *L. jensenii*). Our result also showed the presence of intraspecific variations, which were dispersed in reference gene (**Figure 1A**). The genetic variations were most located in variable region 2 (V2) (33.3%) (**Figure 1B**). Then, to validate whether the novel strains identified among the OTUs were resulted from sequencing errors, we examined their mutations in the supporting reads and their distribution among patients. All 88 *L. iners* OTUs were observed in two or more patients, with at least five supporting full-length 16S rDNA sequences (**Figure 1C**). In order to identify the genetic relationships of the unique strains as well as the existing strains in the database, we draw evolutionary tree showing sequences annotated in the database and sources of the reference data (**Figure 1D**).

In addition, to compare the annotation differences on species levels based on 16S full-length and V3-4 region, we analyzed the top 20 strains. Compared to 16S-FAST, annotation according to V3-4 did not show *L. crispatus*, and OTU was extensively annotated as *L. unclassified* (**Supplementary Figure 4**). This result suggested that the 16S-FAST is indeed required for analysis in cervical microbiota.

### Identification of cervical microbiome type by clustering of samples

**Figure 2:**
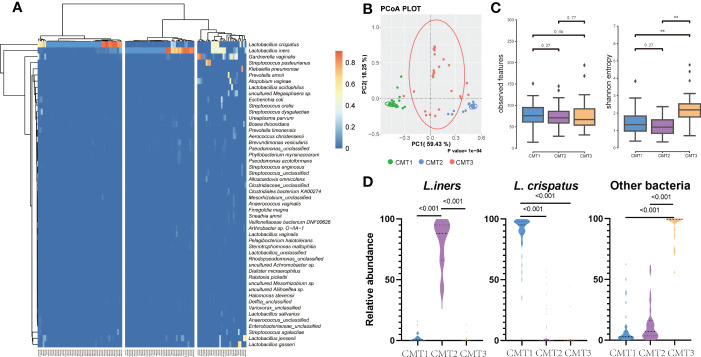
Clustering of cervical bacterial from 120 samples. **(A)** Heatmap of microbial taxa detected in the cervical bacterial communities of 120 participants. A color gradient was used to show abundance. The number of samples is shown on the bottom, and the name of species is shown on the right. Samples were clustered into three groups based on the species composition and abundance of cervical bacterial communities. **(B)** Principal co-ordinates analysis (PCoA) plot between three groups. **(C)** Observed features and Shannon entropy in three groups. **(D)** Relative abundance of *L. crispatus*, *L. iners* and other bacteria in three groups. “Other bacteria” refers to bacteria that is no *L.crisptus* and *L.iners*.

Clustering of the cervical bacterial community was performed at the species level to assess the impact of the cervical microbiome on pregnancy outcomes. According to the 50 most abundant species, the cervical microbiome communities, which we defined as CMTs, were clustered into three major clusters dominated by *L. crispatus* (41.6%) (CMT1), *L. iners* (34.2%) (CMT2), and other bacteria (24.2%) (CMT3) such as *Gardnerella vaginalis, Streptococcus gallolyticus, L. jensenii, Klebsiella pneumoniae, Atopobium vaginae*, *Prevotella amnii, L. gasseri*, and *Streptococcus agalactiae* (**Figure 2A**). β diversity assessed by PCoA separated the three groups (**Figure 2B**). CMT1 had the highest α diversity, followed by CMT2 and CMT3 (**Figure 2C**). The abundance of the main contributors to each CMT strongly avoided each other (**Figure 2D**), suggesting that CMTs were groups of species that contributed to the composition of their microbiome communities.

### Pregnancy outcomes between CMTs

Data of the participants with different CMTs are listed in **Table 1**. Of all participants, 68.3% had a positive biochemical pregnancy, and 59.1% had a positive clinical pregnancy (**Supplementary Table 1**). Among these, CMT1 had significantly higher biochemical and clinical pregnancy rates (**Table 1**). The accompanying distribution of pregnancy outcomes is shown in **Supplementary Figure 5**.

**Table 1:** Basic feature comparison.

		*L. crispatus* dominated group	*L.iners* dominated group	Other bacteria group	
		*N*=50	*N*=41	*N*=29	*P*
Age	<35	37	28	16	0.225
	≥35	13	13	13	
BMI (kg/m^2^)	<24	30	25	20	0.708
	≥24	20	16	9	
Cause of infertility	Tubal factor	31	28	18	1
	Endometriosis	1	2	0	
	Ovarian factor	5	5	1	
	Unknown	4	2	3	
	Male factor	9	4	7	
Endometrial thickness on the day of transplantation (mm)		9.00(8.700-10.00)	9.00(8.50-10.00)	9.30(8.80-10.00)	0.404
Biochemical pregnancy rate (%)		84.00	56.00	58.62	0.008
Clinical pregnancy rate (%)		76.00	46.34	48.28	0.006

Data are presented as number or Median (IQR).

BMI, body mass index.

### Logistic regression analysis of the risk factors for pregnancy failure

To further analyze the risk factors for pregnancy failure, univariate and multivariate logistic regression analyses were conducted to determine the odds ratio (OR) and confidence interval (CI) for the presence of pregnancy negative, which showed that CMT was significantly associated with biochemical pregnancy failure. CMT2 (odds ratio [OR]: 4.109, 95% confidence interval [CI]: 1.549-10.901, *P*=0.05) and CMT3 (OR: 3.706, 95% CI: 1.287-10.667, *P*=0.015) significantly increased the risk factors for biochemical pregnancy failure compared to CMT1. After adjusting for age and embryonic stage as covariates, CMT2 and CMT3 were confirmed as independent risk factors for biochemical pregnancy failure (OR: 6.315, 95% CI: 2.047-19.476, *P*=0.001; OR: 3.635, 95% CI: 1.084-12.189, *P*=0.037). Embryonic stage day 3 was also identified as a risk factor (**Table 2**).

**Table 2:** The logistic analysis of biochemical pregnancy outcome.

		Biochemical pregnancy negative	Biochemical pregnancy positive	*P*	OR (95%CI)	*P*	Adjusted OR (95%CI)	*P*
		*N*=38	*N*=82					
Age	<35	21	60	0.051				
	≥35	17	22		2.208(0.987-4.937)	0.054	1.821(0.723-4.584)	0.203
BMI (kg/m^2^)	<24	22	53	0.478				
	≥24	16	29					
Embryonic stage	cell embryo	15	8	<0.001	6.033(2.270-16.031)	<0.001	8.609(2.759-26.865)	<0.001
	blastocyst	23	74					
Endometrial Thickness on the day of transplantation (mm)		9.85(8.92-11.00)	10.00(8.80-11.45)	0.831				
E_2_ on the day of transplantation (pg/mL)		113.05(68.67-762.14)	104.12(80.29-501.19)	0.856				
Prog on the day of transplantation (ng/mL)		16.98(13.68-21.00)	15.53(13.49-20.84)	0.546				
Cervical community type	*L. crispatus* dominated group	8	42	0.008		**0.010**		**0.006**
	*L.iners* dominated group	18	23		**4.109(1.549-10.901)**	**0.005**	**6.315(2.047-19.476)**	**0.001**
	Other bacteria group	12	17		**3.706(1.287-10.667)**	**0.015**	**3.635(1.084-12.189)**	**0.037**

Data are presented as number or Median (IQR).

BMI, body mass index.

CMT was also significantly associated with clinical pregnancy failure. CMT2 (OR: 3.667, 95% CI: 1.501–8.958, *P*=0.004) and CMT3 (OR: 3.393, 95% CI: 1.279-9.000, *P*=0.014) significantly increased the risk factors for clinical pregnancy failure, and remained independent risk factors for clinical pregnancy failure after adjusted embryonic stage (OR: 4.883, 95% CI: 1.847-12.908, *P*=0.001; OR: 3.478, 95% CI: 1.221–9.911, *P*=0.020) when compared to CMT1 after adjusting for embryonic stage. Embryonic stage day 3 was also identified as a risk factor (**Table 3**).

**Table 3:** The logistic analysis of clinical pregnancy outcome.

		Clinical pregnancy negative	Clinical pregnancy positive	*P*	OR (95%CI)	*P*	Adjusted OR (95%CI)	*P*
		*N*=49	*N*=71					
Age	<35	29	52	0.106				
	≥35	20	19					
BMI (kg/m^2^)		26	49	0.076				
		23	22					
Embryonic stage	cell embryo	16	7	0.002	4.433(1.660-11.841)	0.003	5.779(1.976-16.899)	0.001
	blastocyst	33	64					
Endometrial Thickness on the day of transplantation (mm)		9.90(9.00-11.00)	10.00(8.60-11.25)	0.835				
E_2_ on the day of transplantation (pg/mL)		126.64(68.56-799.57)	102.49(80.42-234.77)	0.626				
Prog on the day of transplantation (ng/mL)		17.25(13.94-22.45)	15.51(13.46-20.06)	0.335				
Cervical community type	*L. crispatus* dominated group	12	38	**0.006**		**0.008**		**0.004**
	*L.iners* dominated group	22	19		**3.667(1.501-8.958)**	**0.004**	**4.883(1.847-12.908)**	**0.001**
	Other bacteria group	15	14		**3.393(1.279-9.000)**	**0.014**	**3.478(1.221-9.911)**	**0.020**

Data are presented as number or Median (IQR).

BMI, body mass index.

### Diagnostic performance of *L. crispatus* for pregnancy outcomes

**Figure 3:**
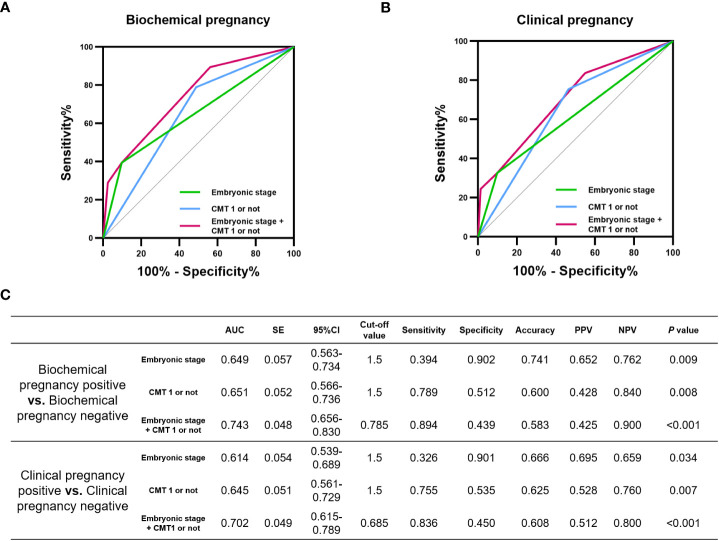
Diagnostic performance of CMT on the incidence of pregnancy failure. Potential of CMT in diagnosing **(A)** biochemical pregnancy failure and **(B)** clinical pregnancy failure. **(C)** area under the curve (AUC), standard error (SE), 95% confidence interval (CI), cut-off value, sensitivity, specificity, Accuracy, positive predictive value (PPV), negative predictive value (NPV) and *P* values for each ROC curve in this evaluation.

We tested the diagnostic performance of CMT by dividing it into CMT1 and non-CMT1. Non-CMT1, as a diagnostic indicator of biochemical pregnancy failure and clinical pregnancy failure, had an area under the curve (AUC) of 0.651 (*P*=0.008) and 0.645 (*P*=0.007), respectively. Additionally, embryonic stage as a diagnostic indicator of biochemical pregnancy failure and clinical pregnancy failure had AUC values of 0.649 (*P*=0.009) and 0.614 (*P*=0.034), respectively. Moreover, combining CMT and embryonic stage optimized the diagnostic performance for biochemical pregnancy failure and clinical pregnancy failure with AUC values of 0.743 (*P*<0.001) and 0.702 (*P*<0.001), respectively (**Figures 3A–C**).

**Figure 4:**
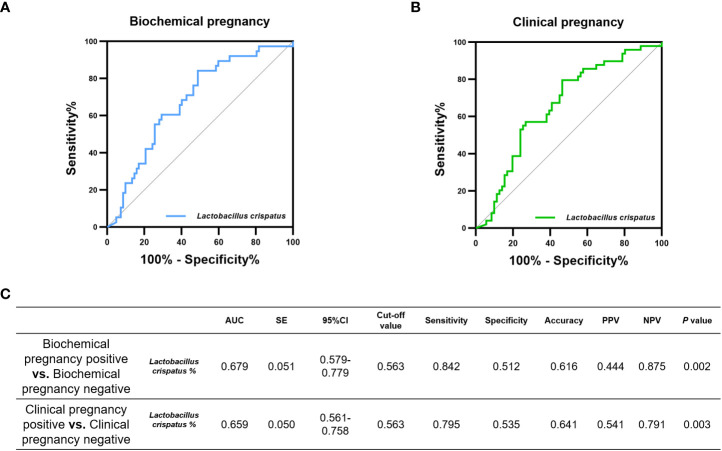
Diagnostic performance of relative abundance of *L.crisptus* measured by 16S-FAST on the incidence of pregnancy outcome. Potential of *L.crisptus* in diagnosing **(A)** biochemical pregnancy failure and **(B)** clinical pregnancy failure. **(C)** area under the curve (AUC), standard error (SE), 95% confidence interval (CI), cut-off value, sensitivity, specificity, Accuracy, positive predictive value (PPV), negative predictive value (NPV) and *P* values for each ROC curve in this evaluation.

In addition, 16S-FAST can accurately identify *L. crispatus*, we also cared about the prediction performance of *L. crispatus* itself on pregnancy outcomes. The relative abundance of *L. crispatus* obtained by 16S-FAST had an AUC of 0.679 (*P*= 0.002) for biochemical pregnancy positive and an AUC of 0.659 (*P*= 0.003) for clinical pregnancy positive (**Figure 4**).

## Discussion

In this study, we used 16S-FAST to describe the characteristics of cervical microbiome of women undergoing IVF, and we evaluated the predictive value of cervical microbiome for IVF outcomes. The cervical microbiome was clustered into three cervical microbiome types (CMT): CMT1, dominated by *L. crispatus*; CMT2, dominated by *L. iners*; and CMT3, dominated by other bacteria. CMT1 had a significantly higher biochemical and clinical pregnancy rate than CMT2 and CMT3. After reducing the bias caused by confounding factors, CMT2 and CMT3 were independent risk factors for biochemical or clinical pregnancy failure. A cervical dominance of *L. crispatus* was an indicator to predict good pregnancy outcomes.

We found *L. crispatus*-dominant microbiome was more prone to pregnancy. Previous studies had suggested that *L. crispatus* was a major composition of reproductive tract bacterial communities and typically had low Nugent scores (Gajer et al., 2012). It could maintain a healthy vaginal microbial ecosystem (Sehring et al., 2022), and was strongly negatively associated with dysbiosis (Borgdorff et al., 2016). Amato et al. observed that a predominance of *L. crispatus* of vaginal microbiome was a marker of a healthy vaginal ecosystem and was associated to intrauterine insemination (IUI) success (Amato et al., 2020). Taken together, infertile women with dominance of *L. crispatus* had a more chance achieving pregnancy success.

Interestingly, in our study, CMT1 and CMT2 were both *Lactobacillus* spp. dominant. However, CMT2 dominant by *L.iners* had not shown a benefit to pregnancy, which was inconsistent with previous research about pregnancy success with *Lactobacillus*. Karaer et al. found that the relative abundance of *Lactobacillus* was lower in women who failed to become pregnant using the V3-V4 regions of the 16S rRNA (Karaer et al., 2021). Bernabeu et al. investigated the vaginal microbiome influences on the IVF outcome by sequencing of V3-V4 region of 16S rRNA, reporting a greater presence of *Lactobacillus* spp. in pregnant women (Bernabeu et al., 2019). But it should be noticed that different species of *Lactobacillus* played different roles in the reproductive tract. Previous researches had shown that the *L. iners*-dominated reproductive tract microbiota appeared to be a risk of increased susceptibility to reproductive tract infections including Chlamydia trachomatis and BV (Macklaim et al., 2011; Santiago et al., 2012; van Houdt et al., 2018; Campisciano et al., 2020; Chen et al., 2022). Lots of researches also indicated that Chlamydia trachomatis and BV led to negative pregnancy outcomes (Wilson et al., 2002; Haahr et al., 2016; Chen et al., 2021a). In addition, traditional 16S rDNA fragment sequencing technology may not be possible to accurately distinguish the species of *Lactobacillus* spp. In this study, we found 16S-FAST improved discrimination ability on species level comparing with traditional sequencing of V3-V4 region of 16S rRNA. Therefore, *Lactobacillus* classification of species level in our study provided a better prediction of a reproductive outcome. In our results, CMT3, including *Gardnerella vaginalis* and *Klebsiella pneumoniae*, which was non-*Lactobacillus* dominant could also be one of risks of lower IVF pregnancy rates, which was consistent with the results of previous study (Sirota et al., 2014; Moreno et al., 2016). Moreno reported that high relative abundances of *Gardnerella vaginalis* and *Klebsiella pneumoniae* were observed in chronic endometritis in asymptomatic infertile women (Moreno et al., 2018), which indicates a negative IVF outcome (Chen et al., 2021b). In addition, non-*Lactobacillus*-predominant microbiota was associated with lower rates of implantation, pregnancy, ongoing pregnancy, live births, and other adverse reproductive outcomes in IVF (Wilson et al., 2002; Sirota et al., 2014; Moreno et al., 2016; Moreno and Simon, 2018).

Unsuccessful outcomes have been linked to dysregulated microbiome in endometrium (Moreno et al., 2022). However, due to the small amount of starting material and low-biomass microbiota, the endometrial microbiota is easily contaminated by exogenous bacterial DNA. Since the microbiome in the upper reproductive tract has been shown to be a continuum (Chen et al., 2017), the cervical microbiome, which is close to the uterine cavity, is convenient to obtain and contamination can be easily prevented. Currently, there are few studies that have reported differences in the cervical microbiome between women with positive assisted reproductive technology (ART) pregnancy outcomes and negative ART outcomes (Hao et al., 2021; Villani et al., 2022). However, the results are controversial and the testing is difficult to apply in clinical working, possibly because it is difficult to accurately detect bacteria at the species level. What’s more, we chose the time of sampling in the endometrial transformation stage, but not on the transfer day, so that we had enough time for testing and advised patients on whether or not to continue the transfer cycle according to the microbiome results. This could be of great benefit in achieving better pregnancy outcomes with interventions for cervical microbiome dysbiosis to postpone IVF and await a favorable profile, such as antibiotics combined with vaginal probiotic therapy (Buggio et al., 2019; Kadogami et al., 2020). A limitation of this study is that a well-defined study population was used, limiting the results to the frozen embryo transfer population. Whether these results can be translated to the general population trying to establish a pregnancy without ART cannot be determined from these data. In addition, this is a cross-sectional study and how cervical microbiome impact the uterine environment for implantation needs further studies.

In summary, we identified and analyzed the cervical microbiome of infertile patients in northern Asia and found that a microbiome dominated by *L. crispatus* was a protective factor for biochemical and clinical pregnancy in IVF patients. On the other hand, *L. iners* and other bacterial-dominated microbiomes were risk factors for pregnancy failure. The cervical community type could be an indicator of pregnancy prediction and provide a more precise treatment plan. However, the reproductive tract microbiome is rich in species and has a high diversity; its interaction with embryo implantation and the maternal host is still unclear, and further exploration and research are required.

## Data availability statement

The data presented in the study are deposited in the NCBI SRA repository, accession number PRJNA903403.

## Ethics statement

The studies involving human participants were reviewed and approved by the Ethics Committee of the Shengjing Hospital of China Medical University. The patients/participants provided their written informed consent to participate in this study.

## Author contributions

WG: study design, execution, manuscript drafting, and critical discussion. SD: study design, execution, analysis, manuscript drafting, and critical discussion. ZW: analysis. JJ: study design, execution, analysis, manuscript drafting, and critical discussion. XW: study design and critical discussion. All authors contributed to the article and approved the submitted version.

## References

[B1] Amato V. Papaleo E. Pasciuta R. Vigano P. Ferrarese R. Clementi N.. (2020). Differential composition of vaginal microbiome, but not of seminal microbiome, is associated with successful intrauterine insemination in couples with idiopathic infertility: a prospective observational study. Open Forum Infect. Dis. 7 (1), ofz525. doi: 10.1093/ofid/ofz525 31915713 PMC6942492

[B2] Bashiri A. Halper K. I. Orvieto R. (2018). Recurrent implantation failure-update overview on etiology, diagnosis, treatment and future directions. Reprod. Biol. Endocrinol. 16 (1), 121. doi: 10.1186/s12958-018-0414-2 30518389 PMC6282265

[B3] Bernabeu A. Lledo B. Diaz M. C. Lozano F. M. Ruiz V. Fuentes A.. (2019). Effect of the vaginal microbiome on the pregnancy rate in women receiving assisted reproductive treatment. J. Assist. Reprod. Genet. 36 (10), 2111–2119. doi: 10.1007/s10815-019-01564-0 31446545 PMC6823330

[B4] Borgdorff H. Armstrong S. D. Tytgat H. L. Xia D. Ndayisaba G. F. Wastling J. M.. (2016). Unique insights in the cervicovaginal lactobacillus iners and l. crispatus proteomes and their associations with microbiota dysbiosis. PloS One 11 (3), e0150767. doi: 10.1371/journal.pone.0150767 26963809 PMC4786256

[B5] Borgdorff H. Tsivtsivadze E. Verhelst R. Marzorati M. Jurriaans S. Ndayisaba G. F.. (2014). Lactobacillus-dominated cervicovaginal microbiota associated with reduced HIV/STI prevalence and genital HIV viral load in African women. ISME J. 8 (9), 1781–1793. doi: 10.1038/ismej.2014.26 24599071 PMC4139719

[B6] Buggio L. Somigliana E. Borghi A. Vercellini P. (2019). Probiotics and vaginal microecology: fact or fancy? BMC Womens Health 19 (1), 25. doi: 10.1186/s12905-019-0723-4 30704451 PMC6357464

[B7] Campisciano G. Iebba V. Zito G. Luppi S. Martinelli M. Fischer L.. (2020). Lactobacillus iners and gasseri, prevotella bivia and HPV belong to the microbiological signature negatively affecting human reproduction. Microorganisms 9 (1), 39. doi: 10.3390/microorganisms9010039 33375526 PMC7824525

[B8] Caporaso J. G. Kuczynski J. Stombaugh J. Bittinger K. Bushman F. D. Costello E. K.. (2010). QIIME allows analysis of high-throughput community sequencing data. Nat. Methods 7 (5), 335–336. doi: 10.1038/nmeth.f.303 20383131 PMC3156573

[B9] Carson S. A. Kallen A. N. (2021). Diagnosis and management of infertility: a review. JAMA 326 (1), 65–76. doi: 10.1001/jama.2021.4788 34228062 PMC9302705

[B10] Chen H. Min S. Wang L. Zhao L. Luo F. Lei W.. (2022). Lactobacillus modulates chlamydia infectivity and genital tract pathology *in vitro* and *in vivo* . Front. Microbiol. 13. doi: 10.3389/fmicb.2022.877223 PMC9098263 35572713

[B11] Chen C. Song X. Wei W. Zhong H. Dai J. Lan Z.. (2017). The microbiota continuum along the female reproductive tract and its relation to uterine-related diseases. Nat. Commun. 8 (1), 875. doi: 10.1038/s41467-017-00901-0 29042534 PMC5645390

[B12] Chen H. Wang L. Zhao L. Luo L. Min S. Wen Y.. (2021a). Alterations of vaginal microbiota in women with infertility and chlamydia trachomatis infection. Front. Cell Infect. Microbiol. 11. doi: 10.3389/fcimb.2021.698840 PMC8370387 34414130

[B13] Chen W. Wei K. He X. Wei J. Yang L. Li L.. (2021b). Identification of uterine microbiota in infertile women receiving *in vitro* fertilization with and without chronic endometritis. Front. Cell Dev. Biol. 9. doi: 10.3389/fcell.2021.693267 PMC8409574 34485281

[B14] Dong S. Jiao J. Jia S. Li G. Zhang W. Yang K.. (2021). 16S rDNA full-length assembly sequencing technology analysis of intestinal microbiome in polycystic ovary syndrome. Front. Cell Infect. Microbiol. 11. doi: 10.3389/fcimb.2021.634981 PMC8141595 34041041

[B15] Edgar R. C. (2004). MUSCLE: multiple sequence alignment with high accuracy and high throughput. Nucleic Acids Res. 32 (5), 1792–1797. doi: 10.1093/nar/gkh340 15034147 PMC390337

[B16] Fu M. Zhang X. Liang Y. Lin S. Qian W. Fan S. (2020). Alterations in vaginal microbiota and associated metabolome in women with recurrent implantation failure. mBio 11 (3), e03242–19. doi: 10.1128/mBio.03242-19 32487762 PMC7267891

[B17] Gajer P. Brotman R. M. Bai G. Sakamoto J. Schutte U. M. Zhong X.. (2012). Temporal dynamics of the human vaginal microbiota. Sci. Transl. Med. 4 (132), 132ra152. doi: 10.1126/scitranslmed.3003605 PMC3722878 22553250

[B18] Haahr T. Jensen J. S. Thomsen L. Duus L. Rygaard K. Humaidan P. (2016). Abnormal vaginal microbiota may be associated with poor reproductive outcomes: a prospective study in IVF patients. Hum. Reprod. 31 (4), 795–803. doi: 10.1093/humrep/dew026 26911864

[B19] Hao X. Li P. Wu S. Tan J. (2021). Association of the cervical microbiota with pregnancy outcome in a subfertile population undergoing *In vitro* fertilization: a case-control study. Front. Cell Infect. Microbiol. 11. doi: 10.3389/fcimb.2021.654202 PMC8495128 34631595

[B20] Hisada T. Endoh K. Kuriki K. (2015). Inter- and intra-individual variations in seasonal and daily stabilities of the human gut microbiota in Japanese. Arch. Microbiol. 197 (7), 919–934. doi: 10.1007/s00203-015-1125-0 26068535 PMC4536265

[B21] Inhorn M. C. Patrizio P. (2015). Infertility around the globe: new thinking on gender, reproductive technologies and global movements in the 21st century. Hum. Reprod. Update 21 (4), 411–426. doi: 10.1093/humupd/dmv016 25801630

[B22] Jespers V. van de Wijgert J. Cools P. Verhelst R. Verstraelen H. Delany-Moretlwe S.. (2015). The significance of lactobacillus crispatus and l. vaginalis for vaginal health and the negative effect of recent sex: a cross-sectional descriptive study across groups of African women. BMC Infect. Dis. 15, 115. doi: 10.1186/s12879-015-0825-z 25879811 PMC4351943

[B23] Kadogami D. Nakaoka Y. Morimoto Y. (2020). Use of a vaginal probiotic suppository and antibiotics to influence the composition of the endometrial microbiota. Reprod. Biol. 20 (3), 307–314. doi: 10.1016/j.repbio.2020.07.001 32680750

[B24] Karaer A. Dogan B. Gunal S. Tuncay G. Arda Duz S. Unver T.. (2021). The vaginal microbiota composition of women undergoing assisted reproduction: a prospective cohort study. BJOG 128 (13), 2101–2109. doi: 10.1111/1471-0528.16782 34053157

[B25] Karst S. M. Dueholm M. S. McIlroy S. J. Kirkegaard R. H. Nielsen P. H. Albertsen M. (2018). Retrieval of a million high-quality, full-length microbial 16S and 18S rRNA gene sequences without primer bias. Nat. Biotechnol. 36 (2), 190–195. doi: 10.1038/nbt.4045 29291348

[B26] Macklaim J. M. Gloor G. B. Anukam K. C. Cribby S. Reid G. (2011). At The crossroads of vaginal health and disease, the genome sequence of lactobacillus iners AB-1. Proc. Natl. Acad. Sci. U.S.A. 108 Suppl 1 (Suppl 1), 4688–4695. doi: 10.1073/pnas.1000086107 21059957 PMC3063587

[B27] Moreno I. Cicinelli E. Garcia-Grau I. Gonzalez-Monfort M. Bau D. Vilella F.. (2018). The diagnosis of chronic endometritis in infertile asymptomatic women: a comparative study of histology, microbial cultures, hysteroscopy, and molecular microbiology. Am. J. Obstet. Gynecol. 218 (6), 602.e601–602.e616. doi: 10.1016/j.ajog.2018.02.012 29477653

[B28] Moreno I. Codoner F. M. Vilella F. Valbuena D. Martinez-Blanch J. F. Jimenez-Almazan J.. (2016). Evidence that the endometrial microbiota has an effect on implantation success or failure. Am. J. Obstet. Gynecol. 215 (6), 684–703. doi: 10.1016/j.ajog.2016.09.075 27717732

[B29] Moreno I. Garcia-Grau I. Perez-Villaroya D. Gonzalez-Monfort M. Bahceci M. Barrionuevo M. J.. (2022). Endometrial microbiota composition is associated with reproductive outcome in infertile patients. Microbiome 10 (1), 1. doi: 10.1186/s40168-021-01184-w 34980280 PMC8725275

[B30] Moreno I. Simon C. (2018). Relevance of assessing the uterine microbiota in infertility. Fertil. Steril. 110 (3), 337–343. doi: 10.1016/j.fertnstert.2018.04.041 30098680

[B31] Nishimoto Y. Mizutani S. Nakajima T. Hosoda F. Watanabe H. Saito Y.. (2016). High stability of faecal microbiome composition in guanidine thiocyanate solution at room temperature and robustness during colonoscopy. Gut 65 (9), 1574–1575. doi: 10.1136/gutjnl-2016-311937 27340192 PMC5036237

[B32] Price M. N. Dehal P. S. Arkin A. P. (2009). FastTree: computing large minimum evolution trees with profiles instead of a distance matrix. Mol. Biol. Evol. 26 (7), 1641–1650. doi: 10.1093/molbev/msp077 19377059 PMC2693737

[B33] Price M. N. Dehal P. S. Arkin A. P. (2010). FastTree 2–approximately maximum-likelihood trees for large alignments. PloS One 5 (3), e9490. doi: 10.1371/journal.pone.0009490 20224823 PMC2835736

[B34] Quast C. Pruesse E. Yilmaz P. Gerken J. Schweer T. Yarza P.. (2013). The SILVA ribosomal RNA gene database project: improved data processing and web-based tools. Nucleic Acids Res. 41 (Database issue), D590–D596. doi: 10.1093/nar/gks1219 23193283 PMC3531112

[B35] Ravel J. Brotman R. M. Gajer P. (2013). Daily temporal dynamics of vaginal microbiota before, during and after episodes of bacterial vaginosis. Microbiome 1 (1), 29. doi: 10.1186/2049-2618-1-29 24451163 PMC3968321

[B36] Reimers L. L. Mehta S. D. Massad L. S. Burk R. D. Xie X. Ravel J.. (2016). The cervicovaginal microbiota and its associations with human papillomavirus detection in HIV-infected and HIV-uninfected women. J. Infect. Dis. 214 (9), 1361–1369. doi: 10.1093/infdis/jiw374 27521363 PMC5079369

[B37] Santiago G. L. Tency I. Verstraelen H. Verhelst R. Trog M. Temmerman M.. (2012). Longitudinal qPCR study of the dynamics of l. crispatus, l. iners, a. vaginae, (sialidase positive) g. vaginalis and p. bivia in the vagina. PloS One 7 (9), e45281. doi: 10.1371/journal.pone.0045281 23028904 PMC3448655

[B38] Schloss P. D. Westcott S. L. Ryabin T. Hall J. R. Hartmann M. Hollister E. B.. (2009). Introducing mothur: open-source, platform-independent, community-supported software for describing and comparing microbial communities. Appl. Environ. Microbiol. 75 (23), 7537–7541. doi: 10.1128/AEM.01541-09 19801464 PMC2786419

[B39] Sehring J. Beltsos A. Jeelani R. (2022). Human implantation: the complex interplay between endometrial receptivity, inflammation, and the microbiome. Placenta 117, 179–186. doi: 10.1016/j.placenta.2021.12.015 34929458

[B40] Sezer O. Soyer Caliskan C. Celik S. Kilic S. S. Kuruoglu T. Unluguzel Ustun G.. (2022). Assessment of vaginal and endometrial microbiota by real-time PCR in women with unexplained infertility. J. Obstet. Gynaecol. Res. 48 (1), 129–139. doi: 10.1111/jog.15060 34657369

[B41] Sirota I. Zarek S. M. Segars J. H. (2014). Potential influence of the microbiome on infertility and assisted reproductive technology. Semin. Reprod. Med. 32 (1), 35–42. doi: 10.1055/s-0033-1361821 24390919 PMC4137456

[B42] van Houdt R. Ma B. Bruisten S. M. Speksnijder A. Ravel J. de Vries H. J. C. (2018). Lactobacillus iners-dominated vaginal microbiota is associated with increased susceptibility to chlamydia trachomatis infection in Dutch women: a case-control study. Sex. Transm. Infect. 94 (2), 117–123. doi: 10.1136/sextrans-2017-053133 28947665 PMC6083440

[B43] Villani A. Fontana A. Barone S. de Stefani S. Primiterra M. Copetti M.. (2022). Identifying predictive bacterial markers from cervical swab microbiota on pregnancy outcome in woman undergoing assisted reproductive technologies. J. Clin. Med. 11 (3), 680. doi: 10.3390/jcm11030680 35160131 PMC8836651

[B44] Wang R. Zhou G. Wu L. Huang X. Li Y. Luo B.. (2021). The microbial composition of lower genital tract may affect the outcome of *in vitro* fertilization-embryo transfer. Front. Microbiol. 12. doi: 10.3389/fmicb.2021.729744 PMC8518997 34659157

[B45] Wilson J. D. Ralph S. G. Rutherford A. J. (2002). Rates of bacterial vaginosis in women undergoing *in vitro* fertilisation for different types of infertility. BJOG 109 (6), 714–717. doi: 10.1111/j.1471-0528.2002.01297.x 12118653

[B46] Witkin S. S. Moron A. F. Linhares I. M. Forney L. J. (2021). Influence of lactobacillus crispatus, lactobacillus iners and gardnerella vaginalis on bacterial vaginal composition in pregnant women. Arch. Gynecol. Obstet. 304 (2), 395–400. doi: 10.1007/s00404-021-05978-z 33521838

[B47] Zhang Y. Xu Y. Wang Y. Xue Q. Shang J. Yang X.. (2021). Comparison of the predictive value of progesterone-related indicators for pregnancy outcomes of women undergoing the short-acting GnRH agonist long protocol: a retrospective study. J. Ovarian Res. 14 (1), 14. doi: 10.1186/s13048-021-00768-2 33436055 PMC7802138

[B48] Zhao C. Wei Z. Yang J. Zhang J. Yu C. Yang A.. (2020). Characterization of the vaginal microbiome in women with infertility and its potential correlation with hormone stimulation during *In vitro* fertilization surgery. mSystems 5 (4), e00450–20. doi: 10.1128/mSystems.00450-20 32665329 PMC7363005

