# Supplementary figures and images for: Impact of a Lactobacillus dominant cervical microbiome, based on 16S-FAST profiling, on the reproductive outcomes of IVF patients
Source: Front Cell Infect Microbiol. 2023 May 26;13:1059339. doi: 10.3389/fcimb.2023.1059339 (PMC10250658; doi:10.3389/fcimb.2023.1059339)

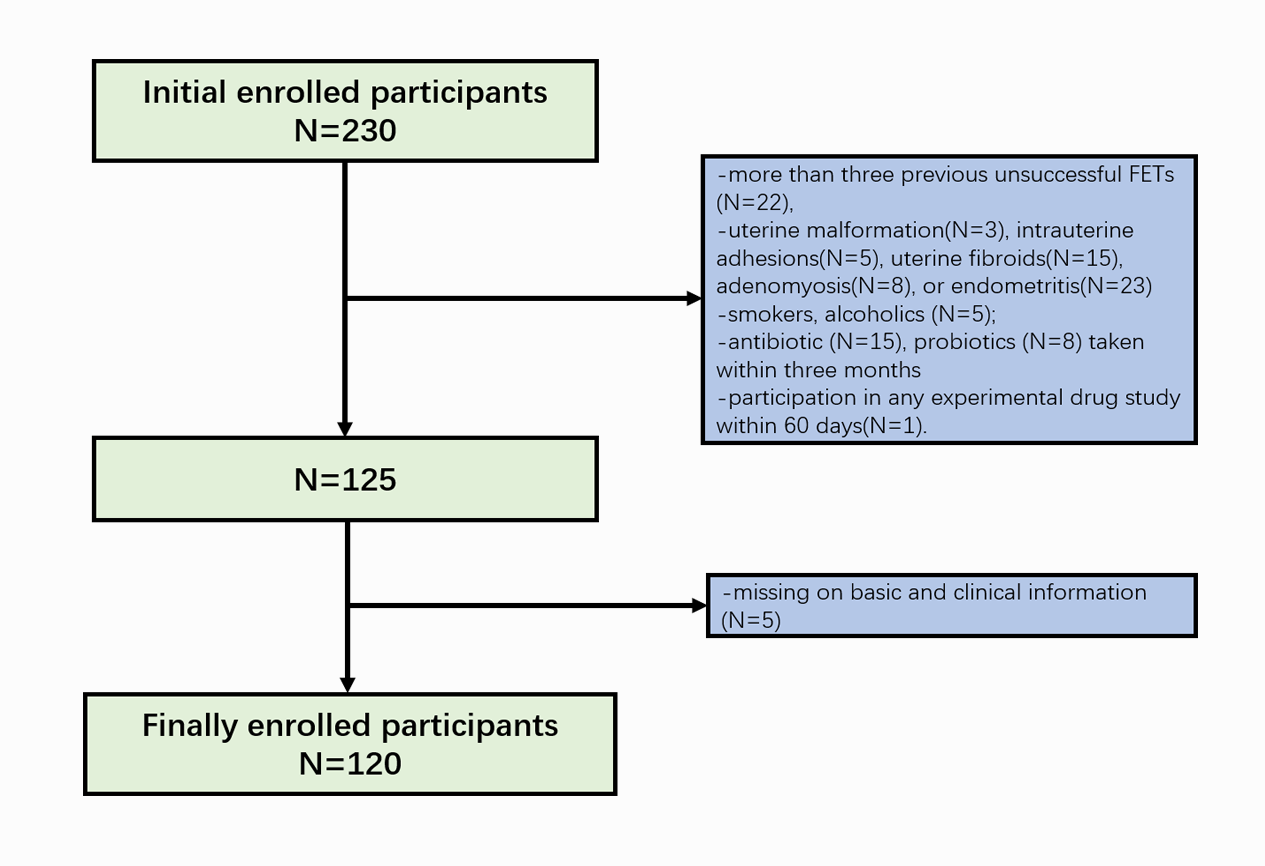

Supplement: Supplementary file 1 [file Image_1.tif]

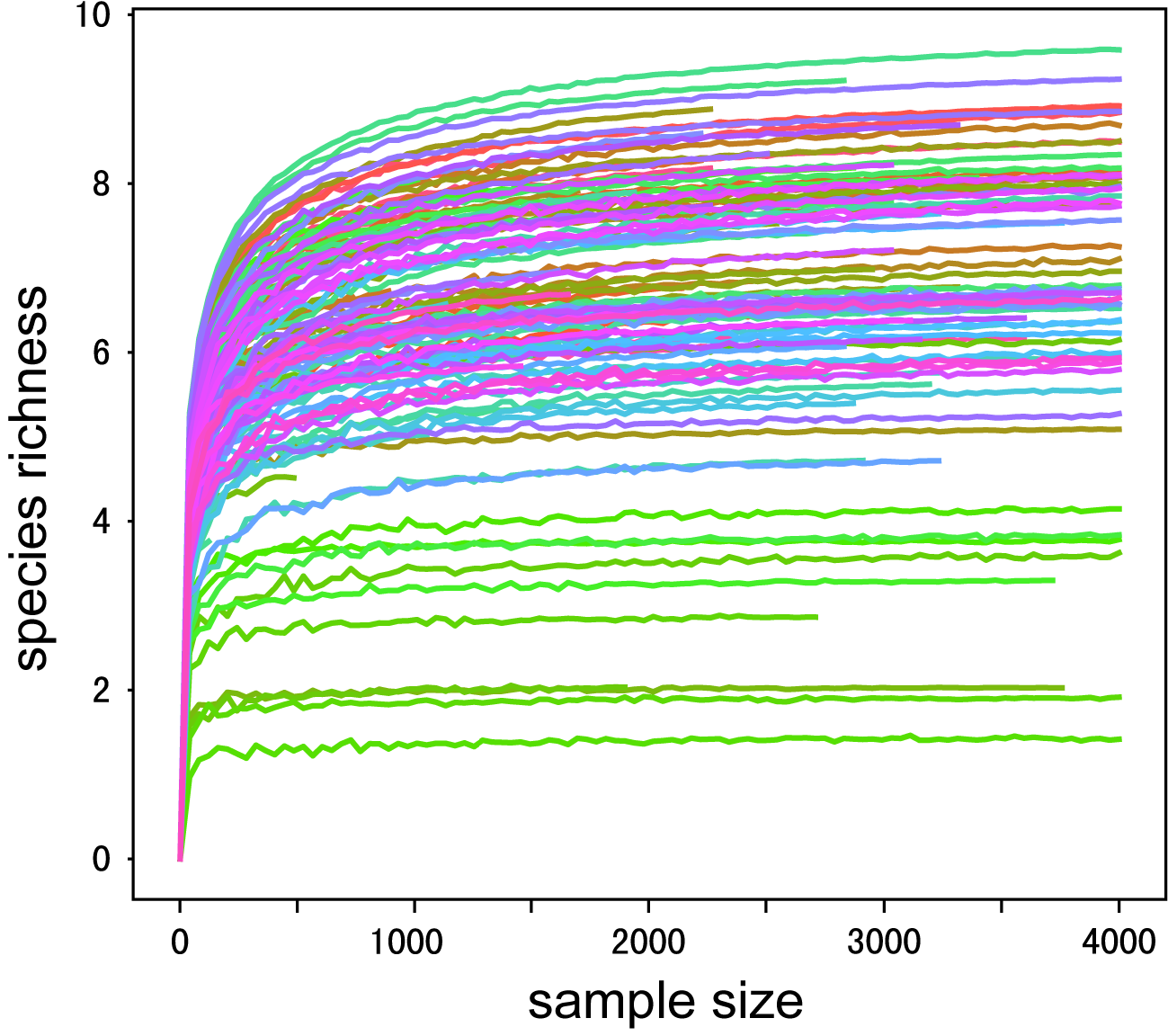

Supplement: Supplementary file 2 [file Image_2.tif]

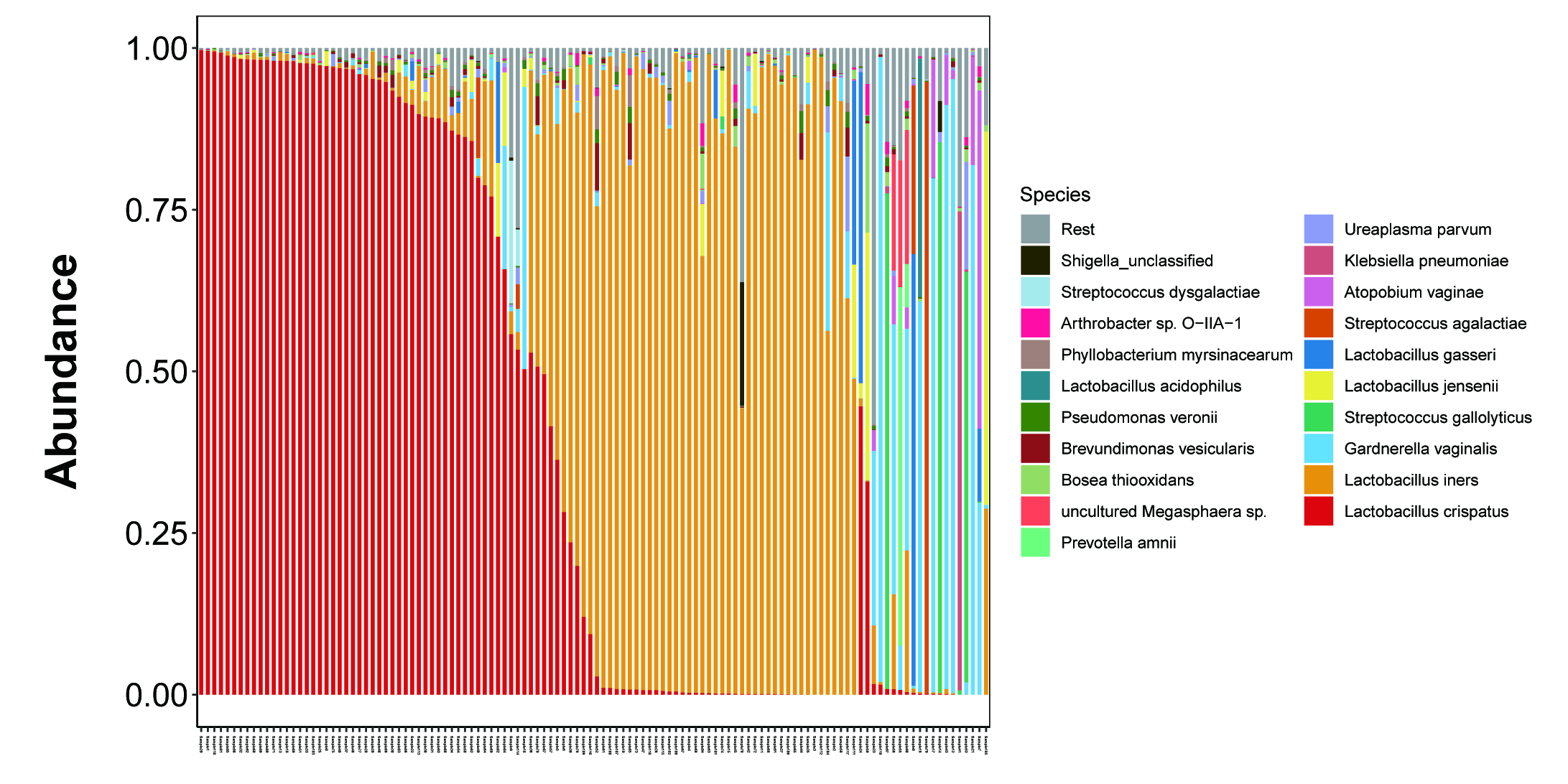

Supplement: Supplementary file 3 [file Image_3.tif]

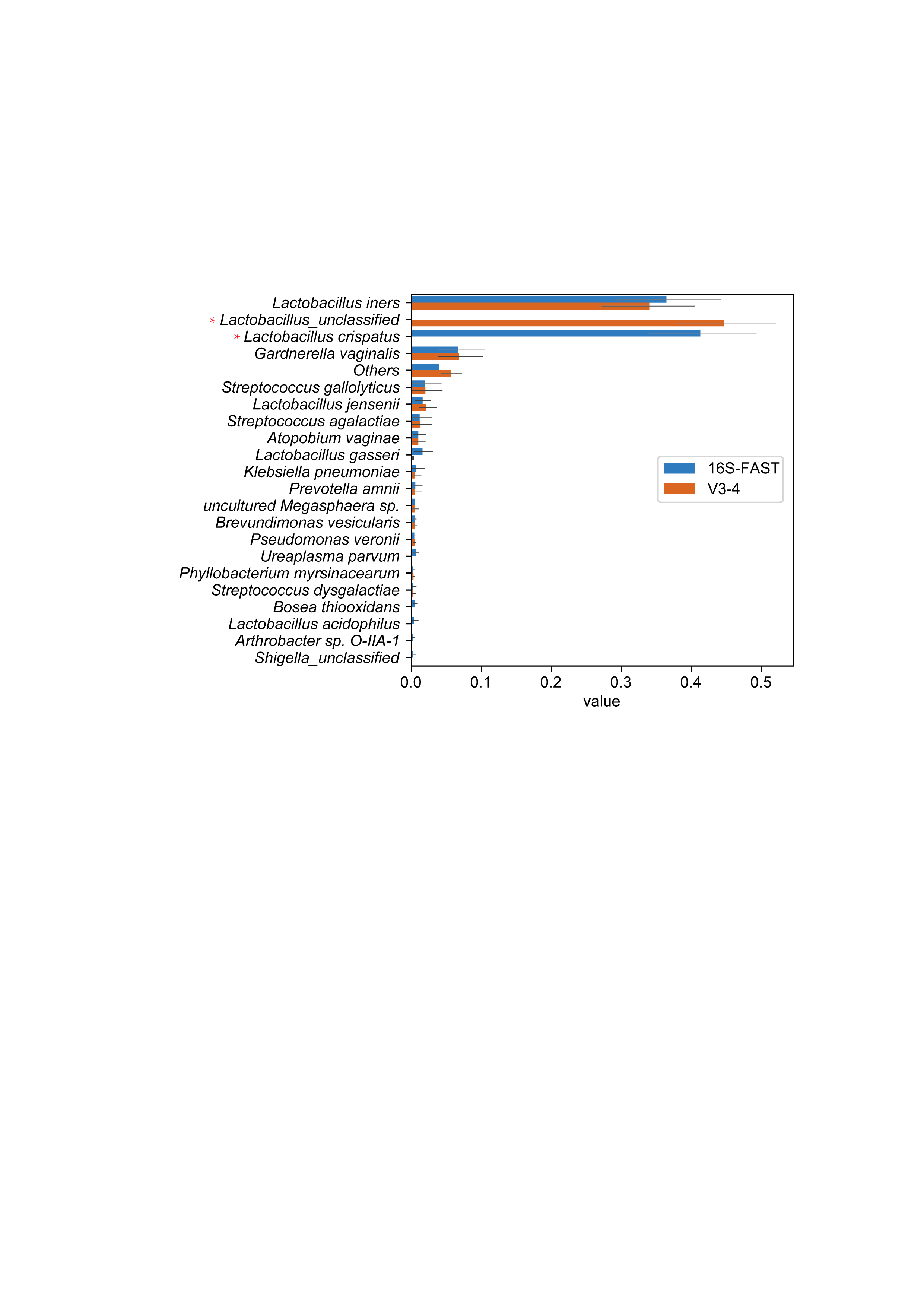

Supplement: Supplementary file 4 [file Image_4.tif]

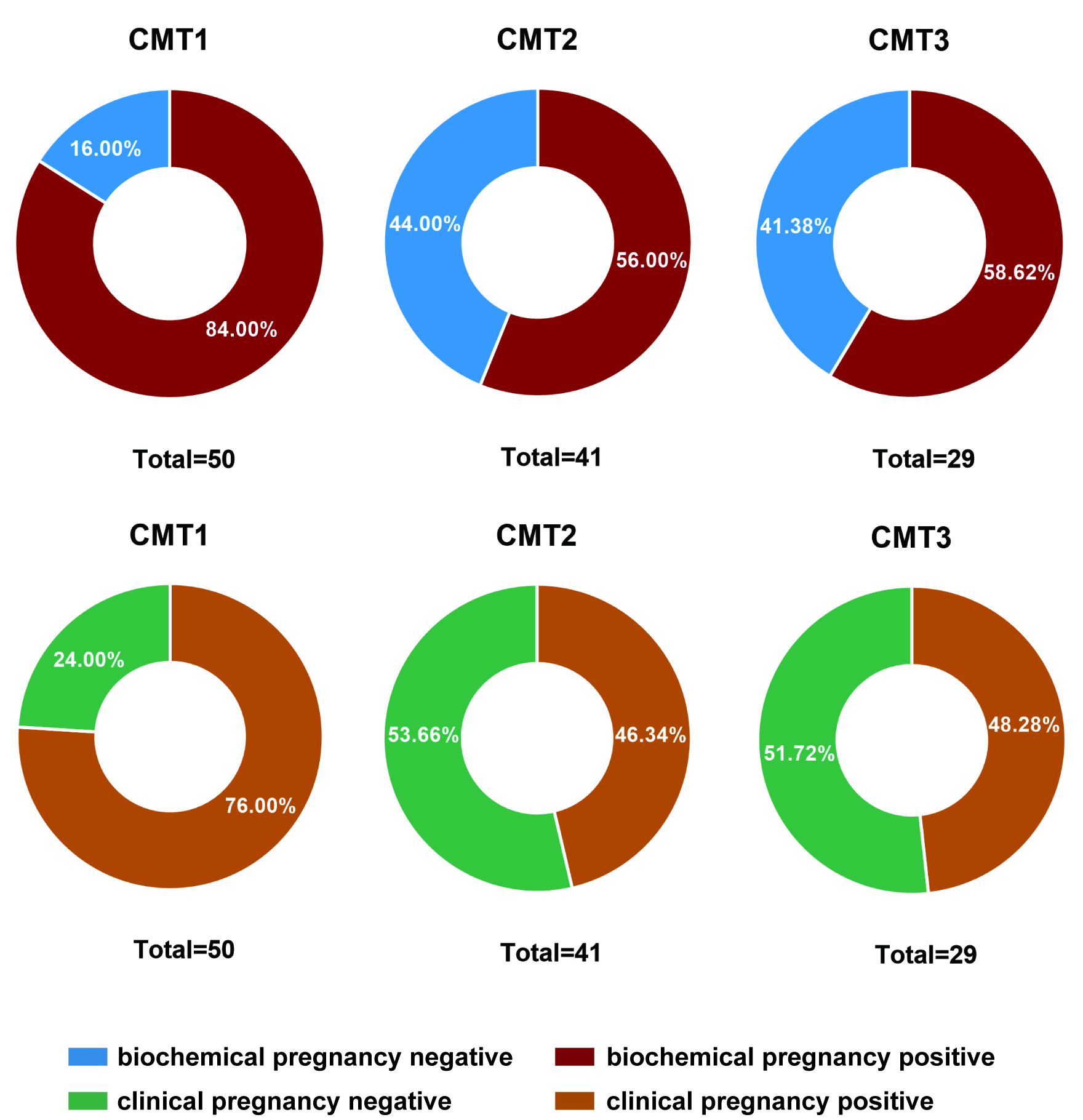

Supplement: Supplementary file 5 [file Image_5.tif]
