# Supplementary material for: Impact of a Lactobacillus dominant cervical microbiome, based on 16S-FAST profiling, on the reproductive outcomes of IVF patients
Source: Front Cell Infect Microbiol. 2023 May 26;13:1059339. doi: 10.3389/fcimb.2023.1059339 (PMC10250658; doi:10.3389/fcimb.2023.1059339)
Supplement: Supplementary file 6 [file Table_1.pdf]

Supplementary Material

Impact of a *Lactobacillus* Dominant Cervical Microbiome, Based on 16S-FAST Profiling, on the Reproductive Outcomes of IVF Patients

Wenzheng Guan*, Sitong Dong*, Zhen Wang

*** Correspondence:**

Jiao Jiao: 13889284796@163.com

Xiuxia Wang: wangxxsj@sina.cn

Supplementary Table

Table S1 Basic characteristics of all participants.

		N=120
Age(*N*)	<35	81
	≥35	39
BMI (kg/m^2) (*N*)	<24	75
	≥24	45
Cause of infertility	Tubal factor	77
	Endometriosis	3
	Ovarian factor	11
	Unknown	9
	Male factor	20
Endometrial thickness on the day of transplantation(mm)		10.00(8.80-11.00)

Biochemical pregnancy rate (%)	68.3
Clinical pregnancy rate (%)	59.1

Data are presented as number or Median (IQR).

BMI, body mass index.
